# Optimized serum stability and specificity of an αvβ6 integrin-binding peptide for tumor targeting

**DOI:** 10.1016/j.jbc.2021.100657

**Published:** 2021-04-16

**Authors:** Ian I. Cardle, Michael C. Jensen, Suzie H. Pun, Drew L. Sellers

**Affiliations:** 1 Department of Bioengineering, University of Washington, Seattle, Washington, USA; 2 Seattle Children's Therapeutics, Seattle, Washington, USA; 3 Department of Pediatrics, University of Washington, Seattle, Washington, USA; 4 Program in Immunology, Fred Hutchinson Cancer Research Center, Seattle, Washington, USA

**Keywords:** cancer therapy, molecular imaging, peptides, integrin, chemical modification, enzymatic stability, cyclization, amino acid substitution, A20FMDV2, αvβ6, ACN, acetonitrile, DFBP, decafluorobiphenyl, DMF, *N*,*N*-dimethylformamide, DPBS, Dulbecco's PBS, EDT, 1,2-ethanedithiol, FBS, fetal bovine serum, FMDV, foot-and-mouth disease virus, RGD, arginine–glycine–aspartate, RTK, arginine–threonine–lysine

## Abstract

The integrin αvβ6 is an antigen expressed at low levels in healthy tissue but upregulated during tumorigenesis, which makes it a promising target for cancer imaging and therapy. A20FMDV2 is a 20-mer peptide derived from the foot-and-mouth disease virus that exhibits nanomolar and selective affinity for αvβ6 *versus* other integrins. Despite this selectivity, A20FMDV2 has had limited success in imaging and treating αvβ6^+^ tumors *in vivo* because of its poor serum stability. Here, we explore the cyclization and modification of the A20FMDV2 peptide to improve its serum stability without sacrificing its affinity and specificity for αvβ6. Using cysteine amino acid substitutions and cyclization by perfluoroarylation with decafluorobiphenyl, we synthesized six cyclized A20FMDV2 variants and discovered that two retained binding to αvβ6 with modestly improved serum stability. Further d-amino acid substitutions and C-terminal sequence optimization outside the cyclized region greatly prolonged peptide serum stability without reducing binding affinity. While the cyclized A20FMDV2 variants exhibited increased nonspecific integrin binding compared with the original peptide, additional modifications with the non-natural amino acids citrulline, hydroxyproline, and d-alanine were found to restore binding specificity, with some modifications leading to greater αvβ6 integrin selectivity than the original A20FMDV2 peptide. The peptide modifications detailed herein greatly improve the potential of utilizing A20FMDV2 to target αvβ6 *in vivo*, expanding opportunities for cancer targeting and therapy.

From 2009 through 2015, pancreatic, liver, lung, and esophageal cancers had the lowest survival rates of any cancer and are projected to contribute to 38% of cancer-related deaths in 2020 (1). Patients are often asymptomatic at early stages with these cancers, preventing timely diagnosis and thereby limiting effective treatment options at later stages of disease. Consequentially, there is a significant need for targeted diagnostics and therapeutics that could identify and treat these cancers at early stages to improve patient outcomes.

Integrins are a family of heterodimeric transmembrane receptors that interact with proteins in the extracellular matrix and on other cells to mediate cell adhesion and migration. While integrins are involved in a variety of healthy biological functions, including embryogenesis, tissue regeneration, and immune cell trafficking (2), their aberrant expression and activity can drive cancer initiation and metastasis (3, 4, 5). Integrins have thus garnered considerable interest as diagnostic and therapeutic targets for cancer (6, 7). One such integrin, αvβ6, is an epithelial-restricted integrin involved in wound healing that has low basal expression in healthy tissue (8). αvβ6 is broadly upregulated in many solid tumor types, including pancreatic (9), liver (10, 11), lung (12, 13), esophageal (14), cervical (15), breast (16), head and neck (17), colon (18), ovarian (19), stomach (20), and oral cancers (21), and its overexpression often correlates with a poor prognosis (22, 23). The role of αvβ6 in tumorigenesis is correspondingly extensive; αvβ6 binds to fibronectin and tenascin for cell adhesion and migration (14, 24, 25), it activates protransforming growth factor beta to promote the epithelial-to-mesenchymal transition (22, 26, 27, 28), and it mediates secretion of matrix metalloproteinases that remodel the extracellular matrix for cancer growth and invasion (18, 29, 30, 31). Given these qualities, the integrin αvβ6 has become the focus of considerable research efforts in the last two decades as a potential target for cancer imaging and therapy (32, 33).

Peptides are attractive targeting ligands for cancer because of their chemical synthesis and small size, enabling inexpensive production, ease of modification, and enhanced solid tumor penetration compared with antibodies (34, 35, 36, 37). A20FMDV2 is a 20-amino acid, arginine–glycine–aspartate (RGD)–containing peptide derived from the G–H loop of the capsid protein viral protein 1 from foot-and-mouth disease virus (FMDV) serotype O_1_ that binds integrin αvβ6 with low nanomolar affinity and high specificity (38, 39, 40). With its favorable binding properties and demonstrated preclinical safety (41, 42), A20FMDV2 has been used in many cancer research applications, including imaging of αvβ6^+^ tumors in mice and humans (40, 43), αvβ6-specific drug delivery *in vitro* and *in vivo* (44), and engineering chimeric antigen receptors for αvβ6-directed adoptive T-cell immunotherapy (45). Recent studies also show the utility of the peptide for imaging idiopathic pulmonary fibrosis and those associated with connective tissue disease, radiation therapy, and severe acute respiratory syndrome coronavirus 2 infection (46, 47, 48).

However, the clinical translation of A20FMDV2 has been limited, in part, by poor metabolic stability of the peptide that impairs its pharmacokinetics (40, 49). Modification of A20FMDV2 with two short PEG chains (∼1 kDa each) reduces peptide degradation and thereby increases tumor retention but also slows peptide clearance from healthy tissue and increases renal retention (50, 51). For these reasons, we sought to engineer an A20FMDV2 peptide with chemistries and amino acid modifications that increase the peptide's inherent metabolic stability. Here, we report the design of cyclized A20FMDV2 variants with selective amino acid modifications and their characterization *in vitro*. We demonstrate that these peptide variants have prolonged stability in serum and retain their binding affinity for αvβ6^+^ cells. Importantly, some of these optimized peptides demonstrate improved αvβ6 specificity over the original A20FMDV2 peptide, further increasing the benefit for future *in vivo* application.

## Results

### Synthesis of decafluorobiphenyl-cyclized A20FMDV2 variants and binding evaluation

**Figure 1:**
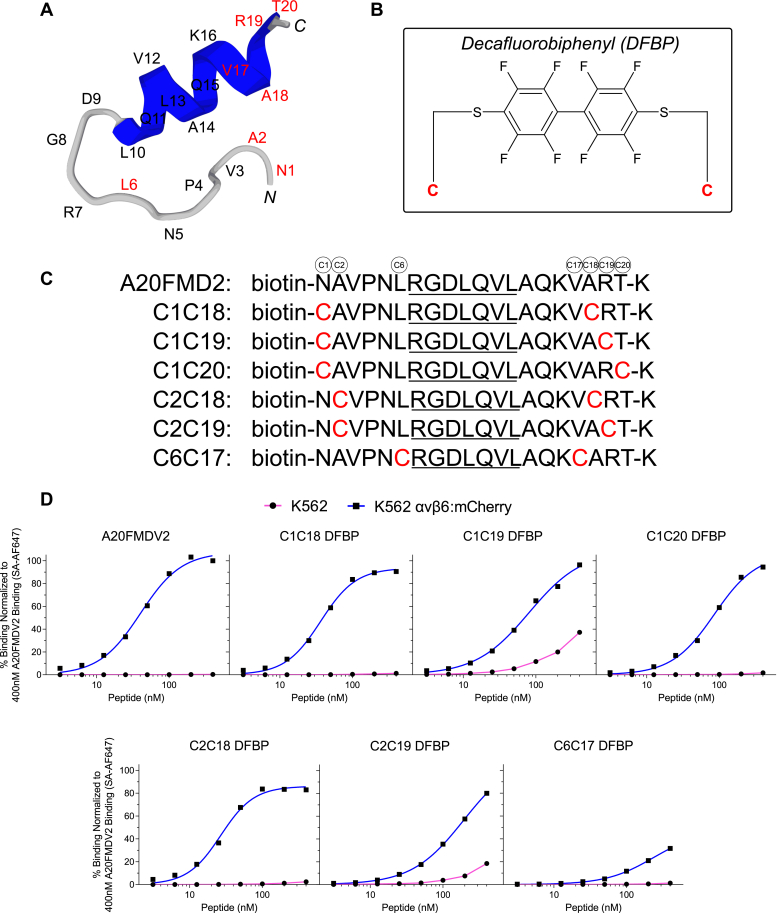
**Site-specific cyclization of A20FMDV2 *via* perfluoroarylation retains peptide binding to αvβ6**^**+**^**cancer cells.***A*, 3D model of A20FMDV2 peptide predicted by PEP-FOLD3 computational framework (68). Amino acids are listed, and positions substituted with cysteines for cyclization are shown in *red*. *B*, chemical structure of decafluorobiphenyl (DFBP) molecular linker used for cyclization. *C*, amino-acid sequences of A20FMDV2 peptide and DFBP-cyclized variants. Cysteine substitutions for cyclization by perfluoroarylation are shown in *red*. The RGDLXXL motif that is important for αvβ6 recognition is *underlined* in all sequences. *D*, flow cytometry binding curves of A20FMDV2 peptide and DFBP-cyclized variants to K562 and K562 αvβ6:mCherry cells, normalized to 400 nM A20FMDV2 binding to K562 αvβ6:mCherry cells. The curves represent a nonlinear regression of one independent experiment in which binding data are fitted to a Hill equation. *K*_*D*_ values are not shown here and will be reported for promising peptides in a later figure with triplicate datasets. SA-AF647, streptavidin Alexa Fluor 647.

A20FMDV2 has a hairpin loop structure with the RGD motif at the tip of hairpin turn followed by a 3_10_ helix (Fig. 1*A*) (38, 39), and previous reports have demonstrated that the extended RGDLXXL motif is most critical for αvβ6 binding, whereas amino acids at the N terminus and C terminus of the peptide are not as critical for binding (52). Accordingly, we postulated that chemistries involving the N- and C-terminal amino-acid positions of the peptide could increase serum stability without negatively affecting peptide binding to αvβ6.

Cyclization is a well-established technique for stabilizing peptides and improving their pharmacokinetic profiles (35), and cysteine perfluoroarylation is a facile cyclization approach that uses perfluoroaromatic molecular linkers to staple together cysteine thiol moieties on unprotected peptides (53). Our laboratory has previously used this technique for peptide cyclization with a decafluorobiphenyl (DFBP) linker (Fig. 1*B*) and demonstrated increased serum stability and affinity of DFBP-cyclized peptides compared with counterparts with disulfide, amide, or triazole cyclization (54, 55). We therefore synthesized six unique A20FMDV2 peptide sequences with cysteine substitutions primarily at N- and C-terminal amino-acid positions for cyclization by DFBP to stabilize and close the hairpin peptide structure (Fig. 1*C*). Biotin was conjugated on the N terminus of all peptides for labeling with streptavidin-AF647 to assess cell binding by flow cytometry. Peptides were also synthesized with a C-terminal lysine to mimic a prospective methyltrityl-lysine that could be added for selective modification or synthesis of branched peptides at the lysine side chain.

The binding of cyclized A20FMDV2 sequences to αvβ6 was evaluated with the matched erythroleukemia K562 and K562 αvβ6:mCherry cell lines. Both cell lines endogenously express the α5β1 integrin (56), but only the K562 αvβ6:mCherry cells express the αvβ6 integrin. Of the DFBP-cyclized sequences, we observed binding of the C1C18 DFBP (N1C; A18C), C1C20 DFBP (N1C; T20C), and C2C18 DFBP (A2C; A18C) peptides to K562 αvβ6:mCherry cells with high affinity and specificity comparable to the original A20FMDV2 peptide (Fig. 1*D*). Interestingly, peptide sequences C1C19 DFBP (N1C; R19C) and C2C19 DFBP (A2C; R19C) exhibited not only high binding to K562 αvβ6:mCherry cells but also poor specificity. Both peptides significantly bound to parental K562 cells at high concentrations, suggesting that Arg19 in A20FMDV2 is important for αvβ6 specificity. Similarly, the peptide cyclized *via* cysteine substitutions proximal to the RGDLXXL motif, C6C17 DFBP (L6C; V17C), displayed minimal binding to K562 αvβ6:mCherry cells. It is known that Val12 and Val17 are important for the structure of the post-RGD helix in A20FMDV2 (57), so the cysteine substitutions in the C6C17 DFBP peptide and their cyclization likely impaired the 3_10_ helix structure. Given that the DFBP-cyclized C1C18, C1C20, and C2C18 peptides retained the favorable binding properties of the original A20FMDV2 peptide, we moved forward with these variants for characterization of serum stability.

### MALDI-TOF MS serum stability of DFBP-cyclized A20FMDV2 variants

**Figure 2:**
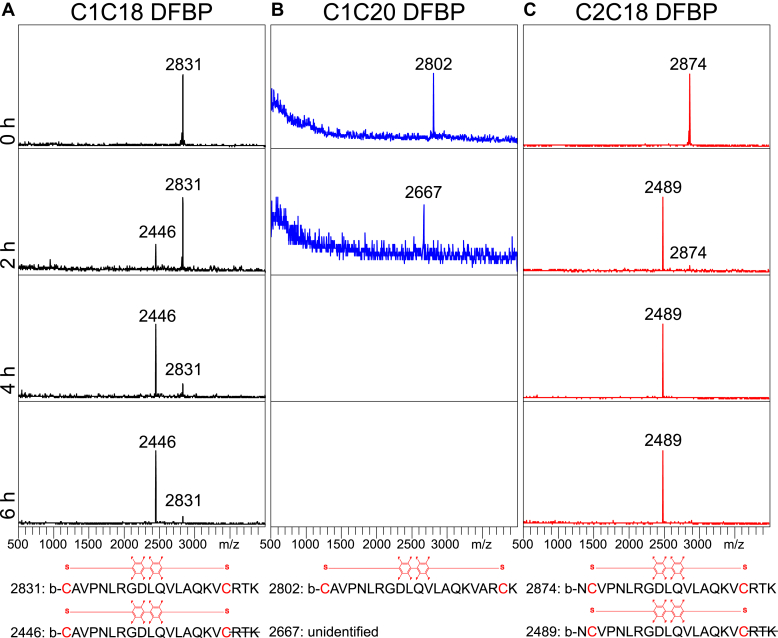
**Partial decafluorobiphenyl (DFBP)-cyclized A20FMDV2 candidates display moderate serum stability with exocyclic C-terminal degradation.***A*–*C*, MALDI-TOF spectra of DFBP-cyclized C1C18, C1C20, and C2C18 peptide variants incubated in normal mouse serum for 0, 2, 4, and 6 h at 37 °C. Molecular weights of prominent peaks are shown. No peptide-fragment peaks were observed for C1C20 DFBP at the 4 and 6 h time points. *Bottom*: predicted amino acid sequences of degradation products based on measured molecular weights.

As the original A20FMDV2 peptide is degraded by over 50% in normal mouse serum within a 4 h incubation at 37 °C (58), we hypothesized that our DFBP-cyclized variants would have prolonged serum stability because of added structural stability from their cyclization. To investigate this, we incubated the DFBP-cyclized C1C18, C1C20, and C2C18 peptides in normal mouse serum at 37 °C for up to 6 h and measured the presence of intact peptide and any degradation products at different time points by MALDI-TOF MS (Fig. 2, *A*–*C*). As shown, the partially cyclized C1C18 DFBP and C2C18 DFBP peptides formed degradation products that are 385 Da smaller after incubation in serum for 2 h, corresponding C-terminal cleavage of the arginine–threonine–lysine (RTK) group outside the DFBP-cyclized region (Fig. 2, *A* and *C*). C1C18 DFBP had prolonged intact peptide presence compared with C2C18 DFBP (6 h *versus* 2 h), suggesting that the C1C18 cyclization scheme better protects the exocyclic C-terminal RTK group. Importantly, the 385 Da smaller degradation products for both peptides persisted up to the last time point sampled, and no further degradation peaks were observed, demonstrating good protection of amino acids within their cyclized regions. We also assayed a disulfide-cyclized C1C18 peptide (C1C18 S–S) and observed faster degradation to the 385 Da smaller product compared with C1C18 DFBP (Fig. S1), emphasizing the importance of the DFBP molecular linker.

To our surprise, the fully cyclized C1C20 DFBP peptide degraded within 2 h in serum; we could not detect any peptide or degradation products at the 4 and 6 h time points (Fig. 2*B*). We detected a low-intensity 135 smaller Da degradation product at the 2 h time point, but it was near background, and we could not predict the amino acid sequence. The molecular weight difference is near what would be expected from an internal arginine deletion (138 Da smaller), so Arg7 or Arg19 may have been metabolically cleaved from the sequence. These data suggest that the complete cyclization of a peptide from N to C terminus is not always beneficial for stability, and that peptide structure should rationally guide cyclization positioning. Since peptides C1C18 DFBP and C2C18 DFBP were stable outside the exocyclic C-terminal RTK groups, we proceeded to further optimize these two peptides with amino acid modifications.

### Modification of DFBP-cyclized C1C18 and C2C18 variants and binding characterization

**Figure 3:**
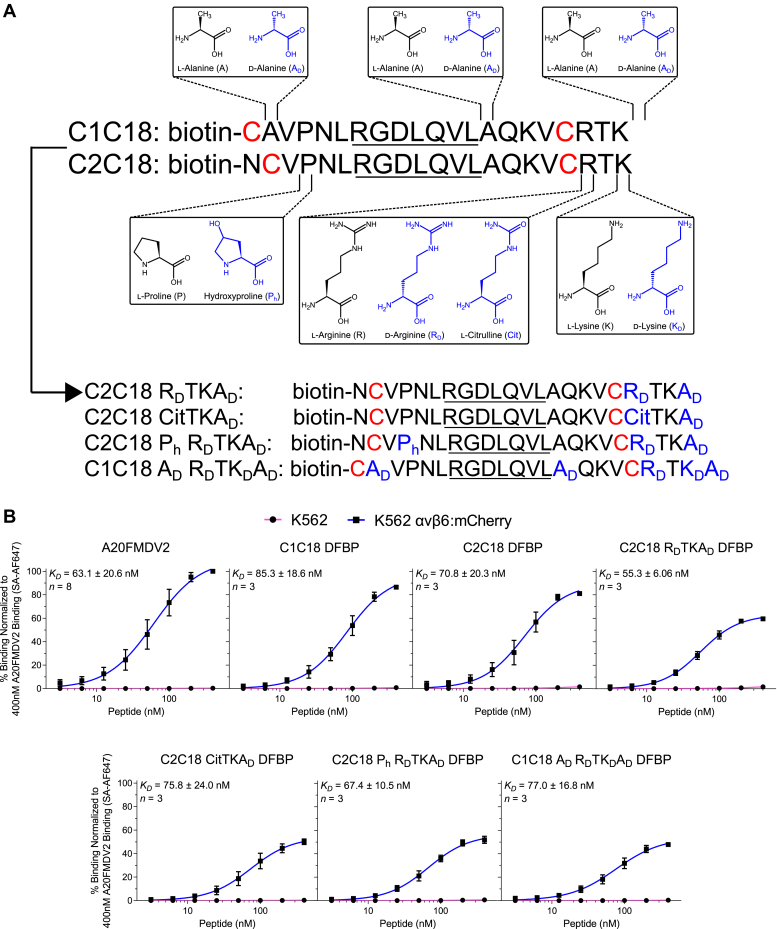
**Further modifications to decafluorobiphenyl (DFBP)-cyclized A20FMDV2 peptides do not impact binding αvβ6**^**+**^**cancer cells.***A*, schematic of modifications made to the sequences of C1C18 DFBP and C2C18 DFBP to further improve their serum stability. Chemical structures of the original (*black*) and modified (*blue*) amino acids are shown at positions of incorporation. The resulting modified peptide sequences are also listed, with cysteine substitutions for DFBP cyclization shown in *red* and amino acid modifications shown in *blue*. The RGDLXXL motif that is important for αvβ6 recognition is *underlined* in all sequences. *B*, flow cytometry binding curves of A20FMDV2, C1C18 DFBP, C2C18 DFBP, and additionally modified peptides to K562 and K562 αvβ6:mCherry cells, normalized to 400 nM A20FMDV2 binding to K562 αvβ6:mCherry cells. The curves represent a nonlinear regression of at least three independent experiments in which binding data are fitted to a Hill equation. *K*_*D*_ values were calculated by averaging the individual regression values of the independent experiments. Data points, error bars, and *K*_*D*_ values represent the mean ± SD; n = 3 to 8 independent experiments. *K*_*D*_ values of cyclized and modified peptides were not statistically different than that of the original peptide (*p* > 0.05, one-way ANOVA with Dunnett's test) and each other (*p* > 0.05, one-way ANOVA with Tukey's test). SA-AF647, streptavidin Alexa Fluor 647.

To further stabilize the DFBP-cyclized C1C18 and C2C18 peptides against proteolytic degradation, we turned to nonproteinogenic d-amino acids and naturally occurring analogs for additional amino acid modification (Fig. 3*A*). As other groups have shown increased serum stability of peptides flanked with d-amino acids (59), we capped the C terminus of our peptides with d-alanine (A_D_) in an effort to reduce degradation of the exocyclic C-terminal RTK group. Moreover, as the exocyclic C-terminal arginine is readily cleaved by many endopeptidases (60), we substituted it with d-arginine (R_D_) or the nonprotein precursor l-citrulline (Cit) as a way to further limit degradation. These peptide modifications resulted in the synthesis of DFBP-cyclized C2C18 R_D_TKA_D_ (R19R_D_; +22A_D_) and C2C18 CitTKA_D_ (R19Cit; +22A_D_) for testing. We also attempted to enhance the binding kinetics of our cyclized peptides by substituting the N-terminal proline within the cyclized region with hydroxyproline (P_h_), since this modification has previously demonstrated affinity improvements for other peptides (61). This led to the synthesis of DFBP-cyclized C2C18 P_h_ R_D_TKA_D_ (P4P_h_; R19R_D_; +22A_D_) for comparison against C2C18 R_D_TKA_D_ DFBP, which lacks the hydroxyproline group. Finally, as the alanines within the cyclized region and the exocyclic C-terminal lysine likely do not contribute to peptide binding, we substituted these amino acids with d-alanine and d-lysine (K_D_), respectively, for additional proteolytic stability. Accordingly, DFBP-cyclized C1C18 A_D_ R_D_TK_D_A_D_ (A2A_D_; A14A_D_; R19R_D_; K21K_D_; +22A_D_) was created to reflect these changes. In total, four modified versions of either DFBP-cyclized C1C18 or C2C18 were synthesized for subsequent binding characterization.

As detailed previously, we compared the K562 cell binding of these newly modified variants against the original A20FMDV2 peptide and the parental DFBP-cyclized C1C18 and C2C18 peptides. As shown, all DFBP-cyclized modified peptides retain selective binding to K562 αvβ6:mCherry cells with negligible background binding to K562 parental cells (Fig. 3*B*). Notably, the apparent binding affinities of these modified peptides (C2C18 R_D_TKA_D_ DFBP: 55.3 ± 6.06 nM; C2C18 CitTKA_D_ DFBP: 75.8 ± 24.0 nM; C2C18 P_h_ R_D_TKA_D_ DFBP: 67.4 ± 10.5 nM; and C1C18 A_D_ R_D_TK_D_A_D_ DFBP: 77.0 ± 16.8 nM) as well as those of the parental DFBP-cyclized C1C18 and C2C18 peptides (85.3 ± 18.6 and 70.8 ± 20.3 nM, respectively), did not statistically differ from the affinity of the original A20FMDV2 peptide (63.1 ± 20.6 nM). We did, however, observe less maximal fluorescence signal for the newly modified peptides compared with A20FMDV2, C1C18 DFBP, and C2C18 DFBP. Given their similar affinities, we attributed these differences to reduced fluorescence quantum yield or biotin accessibility of the streptavidin Alexa Fluor 647 stain. Also of importance, the binding affinities observed here differ from the single digit nanomolar values reported for A20FMDV2 previously (41). These discrepancies likely stem from differences in the binding model (cells *versus* purified recombinant proteins) and conditions (buffer, temperature, and time) used for peptide characterization.

There were no obvious differences when comparing the larger effects of the cyclization schemes and modifications on binding affinity. DFBP-cyclized C1C18 and C2C18 peptides exhibited equivalent apparent binding affinity for K562 αvβ6:mCherry cells, and further stabilizing the C terminus with d-arginine, citrulline, d-lysine, and/or d-alanine did not impact affinity. Interestingly, substituting proline for hydroxyproline and alanine for d-alanine within the cyclized region did not impact apparent binding affinity, suggesting that these amino acids are not critical for high-affinity αvβ6 binding. The effects of these cyclization schemes and modifications on peptide specificity for the αvβ6 integrin will be evaluated in a later section. Considering that all the newly modified DFBP-cyclized peptides retained their binding affinity for αvβ6^+^ cells, we advanced forward with these peptides for qualitative and quantitative assessments of serum stability.

### MALDI-TOF MS serum stability of additionally modified C1C18 and C2C18 DFBP

**Figure 4:**
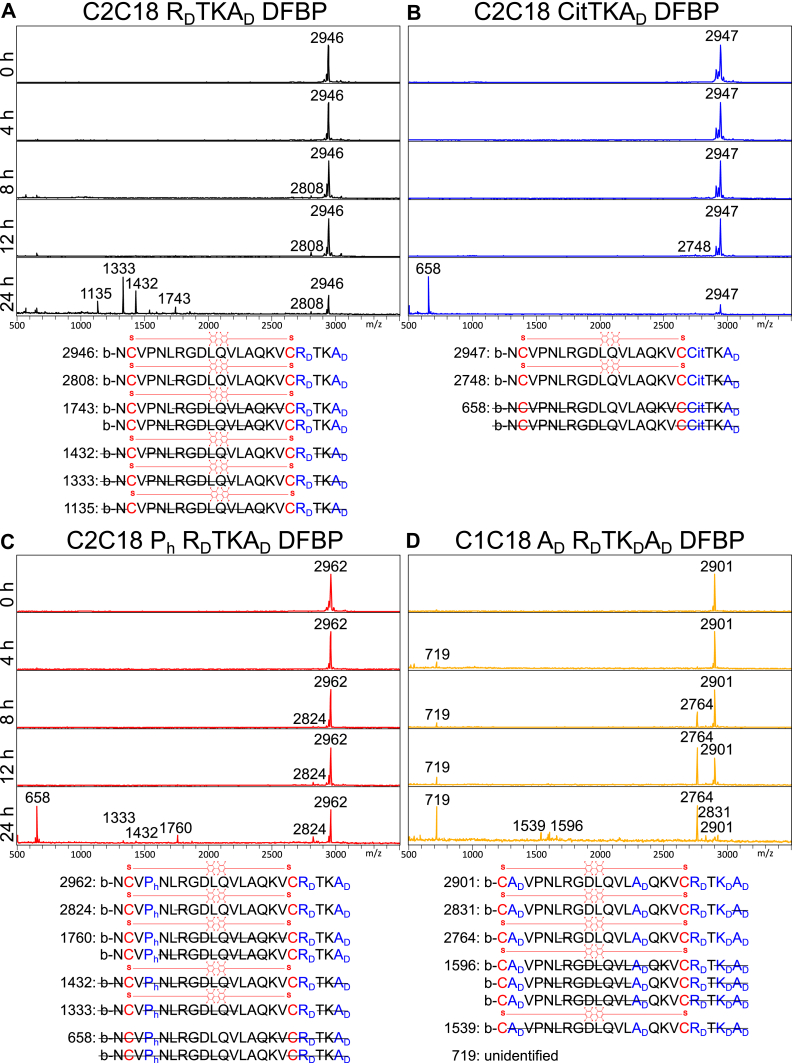
**Further modified decafluorobiphenyl (DFBP)-cyclized A20FMDV2 peptides exhibit prolonged serum stability with slow internal arginine cleavage.***A–D*, MALDI-TOF spectra of DFBP-cyclized C2C18 R_D_TKA_D_, C2C18 CitTKA_D_, C2C18 P_h_ R_D_TKA_D_, and C1C18 A_D_ R_D_TK_D_A_D_ peptides incubated in normal mouse serum for 0, 4, 8, 12, and 24 h at 37 °C. Molecular weights of prominent peaks are shown. *Bottom*: predicted amino acid sequences of degradation products based on measured molecular weights. For some molecular weights, multiple predictions are listed.

We next qualitatively assessed the serum stability of the newly modified peptides by MALDI-TOF MS. Fully intact peptide was still present after 24 h for each of the newly modified peptides (Fig. 4, *A*–*D*), a large improvement compared with the original DFBP-cyclized C1C18 and C2C18 peptides that were degraded completely within 6 h. Nonetheless, degradation products were still detected at later time points, and the relative intact peptide signal decreased over time, an indication of slow degradation of each of the peptides. Interestingly, Arg7 of the RGD motif appeared to be a primary target/site for proteolytic cleavage, as a 138 Da smaller degradation product was seen after 8 h for C2C18 R_D_TKA_D_ DFBP, C2C18 P_h_ R_D_TKA_D_ DFBP, and C1C18 A_D_ R_D_TK_D_A_D_ DFBP (Fig. 4, *A*, *C*, and *D*). Many degradation products at 24 h show peptides missing even larger fragments between the cysteine linkages, suggesting that the initial Arg7 cleave accelerated endopeptidase activity. In addition, the relative signal of the 138 Da smaller degradation product increased over time for C1C18 A_D_ R_D_TK_D_A_D_ DFBP (Fig. 4*D*), which may signify that the C1C18 cyclization scheme better prevents further internal degradation than the C2C18 configuration after initial Arg7 cleavage. Outside the cyclized region, we also observed N-terminal cleavage of biotinylated Asn1 for some of the modified C2C18 peptides (Fig. 4, *A* and *C*) as well as C-terminal cleavage at Thr20 for all the modified peptides (Fig. 4, *A*–*D*). While further peptide design addressing these observed cleavage products warrants future research, the presence of intact cyclized peptides after 24 h in serum demonstrates markedly improved serum stability.

### LC–MS serum stability of additionally modified C1C18 and C2C18 DFBP

**Figure 5:**
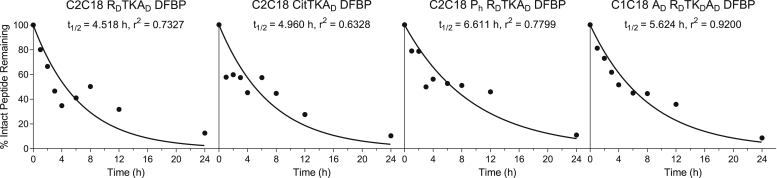
**Further modified decafluorobiphenyl (DFBP)-cyclized A20FMDV2 peptides have long and comparable serum half-lives.** Stability of DFBP-cyclized C2C18 R_D_TKA_D_, C2C18 CitTKA_D_, C2C18 P_h_ R_D_TKA_D_, and C1C18 A_D_ R_D_TK_D_A_D_ peptides over a 24-h incubation in normal mouse serum, as measured by LC–MS. Values are normalized to the 0 h time point. Curves represent a nonlinear regression of one independent experiment assuming one-phase exponential decay.

Since MALDI-TOF MS results are qualitative, we next sought to quantify the rate of degradation in normal mouse serum of the modified DFBP-cyclized C2C18 R_D_TKA_D_, C2C18 CitTKA_D_, C2C18 P_h_ R_D_TKA_D_, and C1C18 A_D_ R_D_TK_D_A_D_ peptides by LC–MS. Peptides were prepared, treated in serum, and extracted as they were for the MALDI-TOF MS studies before submission for LC–MS analysis. While some of the data points are erratic because of variability in peptide extraction after acetonitrile (ACN) precipitation of serum proteins, we were able to reasonably fit the LC–MS results to a one-phase exponential decay model (Fig. 5). Consistent with the MALDI results, the DFBP-cyclized and additionally modified peptides were found to degrade slowly in serum, with serum half-lives of intact peptides stretching between 4.5 and 6.6 h. After 24 h, the amount of remaining fully intact peptide in serum for DFBP-cyclized C2C18 R_D_TKA_D_, C2C18 CitTKA_D_, C2C18 P_h_ R_D_TKA_D_, and C1C18 A_D_ R_D_TK_D_A_D_ ranged tightly at 12.6%, 10.4%, 10.9%, and 8.6%, respectively, demonstrating that they have similar basal stabilities. These results broadly suggest that citrulline and d-arginine substitutions at the peptide C terminus (R19Cit and R19R_D_, respectively) confer comparable proteolytic stabilities, whereas substitutions with hydroxyproline and d-alanine in the cyclized region (P4P_h_, A2A_D_, and A14A_D_, respectively) have negligible impact on intact peptide serum stability.

For C1C18 A_D_ R_D_TK_D_A_D_ DFBP specifically, LC–MS also revealed significant accumulation of the 138 Da smaller degradation product that was identified previously by MALDI-TOF (Fig. S2). After 12 h in serum, the amount of the 138 Da smaller degradation product peaked, representing nearly 60% of the starting (*t* = 0 h) fully intact peptide; even after 24 h in serum, the presence of the degradation product was still significant, representing over 40% of the starting fully intact peptide. These data indicate that internal cleavage of Arg7 from the RGD motif is a primary degradation pathway for these modified peptides and that the resulting product remains relatively stable for the C1C18 A_D_ R_D_TK_D_A_D_ DFBP formulation despite the cleavage. Thus, even more stable cyclized peptides could be potentially made by further modifying the Arg7 in the RGD motif.

### Assessment of arginine mimetics for modification of the RGD motif

Given the previous observations, we were interested in substituting arginine within the RGD motif with nonproteinogenic mimetics to prolong the already enhanced stability of our modified DFBP-cyclized A20FMDV2 variants. Phenylalanine analogs have previously demonstrated to be good mimetics for arginine replacement in other peptides (62). We therefore synthesized three versions of DFBP-cyclized C2C18 containing either 4-amino-l-phenylalanine (_A_F), citrulline, or 4-guanidino-l-phenylalanine (_G_F) as substitutions for Arg7 within the RGD motif (Fig. S3*A*). These peptides were, respectively, named C2C18 _A_FGD RTKA_D_ DFBP, C2C18 CitGD DFBP, and C2C18 _G_FGD R_D_TKA_D_ DFBP. The three peptides have varying degrees of C-terminal modifications, as some of these peptides were synthesized before we discovered the benefit of having d-amino acids at the C terminus. We assumed these differences would not affect our evaluation of binding potential since we showed earlier that these modifications do not influence peptide affinity for αvβ6.

C2C18 _A_FGD RTKA_D_ DFBP and C2C18 _G_FGD R_D_TKA_D_ DFBP completely lost the ability to bind K562 αvβ6:mCherry cells, whereas C2C18 CitGD DFBP retained some selective binding to these cells at high concentrations, albeit low and nonsaturating (Fig. S3*B*). Owing to the highly conserved nature of the RGD motif in integrin binding, these negative results are not surprising. Other groups have also reported drastic loss of binding for an αvβ3-specific peptide when replacing arginine in the RGD motif with closely resembling homologs (63). Nonetheless, the small binding we observed with a citrulline substitution is promising, warranting future investigation with a more expansive panel of mimetics and analogs.

### Nonspecific binding of DFBP-cyclized and modified A20FMDV2 peptides to A375P cells

As there are eight known integrins that recognize the RGD motif (αvβ1, αvβ3, αvβ5, αvβ6, αvβ8, α5β1, α8β1, and αIIbβ3) (64), it is critical that RGD-containing peptides developed for diagnostic and therapeutic applications are highly specific for their targeted integrin with minimal off-target binding. The matched K562 cell model we used previously only expresses the α5β1 integrin and thus does not rigorously test the nonspecific binding of our engineered A20FMDV2 peptides to other RGD-recognizing integrins closely related to αvβ6. Accordingly, we next sought to compare the nonspecific integrin binding of our serum-stabilized peptides against the original A20FMDV2 peptide and the parental DFBP-cyclized C1C18 and C2C18 peptides to melanoma A375P cells, which express the αvβ3, αvβ5, αvβ8, and α5β1 integrins but not the β6 integrin (65). We decided to use A375P cells as a proxy to distinguish how our peptide cyclizations and modifications affect promiscuous integrin binding. To this end, we evaluated binding of A20FMDV2 and DFBP-cyclized C1C18, C2C18, C2C18 R_D_TKA_D_, C2C18 CitTKA_D_, C2C18 P_h_ R_D_TKA_D_, and C1C18 A_D_ R_D_TK_D_A_D_ to A375P cells at four high peptide concentrations (125, 250, 500, and 1000 nM) by flow cytometry.

**Figure 6:**
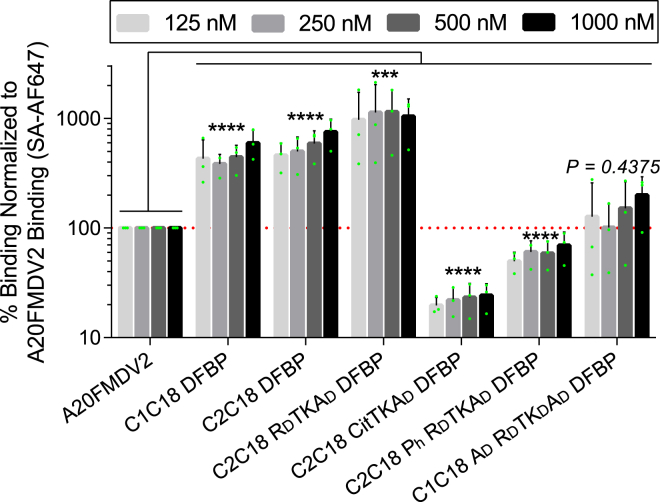
**Citrulline, hydroxyproline, and****d****-alanine substitutions reduced nonspecific integrin binding of decafluorobiphenyl (DFBP)-cyclized A20FMDV2 peptides.** Nonspecific binding of DFBP-cyclized and modified peptides to A375P cells by flow cytometry, normalized to 125, 250, 500, and 1000 nM A20FMDV2 binding. Values that fall above the *dotted red line* represent increased nonspecific binding compared with the original A2FMDV2 peptide, whereas those that fall below represent decreased nonspecific binding. The *green dots* represent the data from individual experiments. Columns and error bars represent the mean ± SD; n = 3 independent experiments. For statistical testing, values from independent experiments and all four concentrations were pooled together for each peptide before comparison to the original peptide (n = 12, *p* > 0.05, *∗∗∗p* < 0.001, *∗∗∗∗p* < 0.0001, paired one-way ANOVA with Dunnett's test). SA-AF647, streptavidin Alexa Fluor 647.

We demonstrate that DFBP-cyclized C1C18 and C2C18 exhibit high nonspecific binding for A375P cells, with an average 462 ± 158% and 575 ± 196% binding, respectively, relative to the original A20FMDV2 peptide across all four concentrations (Fig. 6). These results are consistent with findings made by Wagstaff *et al*. (66), which showed that disulfide cyclization of the A20FMDV2 peptide at certain positions altered the backbone dynamics of the peptide, driving loss of αvβ6 specificity. The only exception that they found not to affect A20FMDV2 specificity was a complete N-to-C terminal disulfide cyclization, but we demonstrated earlier that this cyclization scheme likely underwent rapid degradation in serum (Fig. 2*B*, C1C20 DFBP). Interestingly, even higher nonspecific binding was shown for C2C18 R_D_TKA_D_ DFBP (1072 ± 612% on average relative to A20FMV2), suggesting that C-terminal substitution with d-arginine (R19R_D_) has structural effects that aggravate promiscuous integrin binding.

Most notably, DFBP-cyclized C2C18 CitTKA_D_, C2C18 P_h_ R_D_TKA_D_, and C1C18 A_D_ R_D_TK_D_A_D_ peptides did not display high nonspecific binding, showing on average 22 ± 6%, 60 ± 16%, 145 ± 96% binding relative to the original A20FMDV2 peptide, respectively, across all four concentrations. While some of these effects may be attributed to the reduced binding fluorescence we observed previously with these peptides (∼50%, Fig. 3*B*), it does not completely account for their low nonspecific binding, especially for C2C18 CitTKA_D_ DFBP. Furthermore, the C2C18 R_D_TKA_D_ DFBP peptide has high nonspecific binding despite also exhibiting reduced binding fluorescence (∼60%, Fig. 3*B*), so these two observations do not always track with each other. These data thus suggest that the electrostatic effects of the citrulline substitution (Cit19R_D_), the hydrogen bonding or stereoelectronic effects of the hydroxyproline substitution (P4P_h_), and the structural effects of the d-alanine substitutions (A2A_D_ and A14A_D_) offset the high nonspecific integrin binding observed for the DFBP-cyclized C1C18, C2C18, and C2C18 R_D_TKA_D_ peptides, with some (Cit19R_D_) instilling even improved αvβ6 integrin specificity compared with the original A20FMDV2 peptide. Combined with their greatly enhanced serum stability, the DFBP-cyclized C2C18 CitTKA_D_, C2C18 P_h_ R_D_TKA_D_, and C1C18 A_D_ R_D_TK_D_A_D_ peptides demonstrate the potential to increase αvβ6 targeting for high-fidelity *in vivo* diagnostic and therapeutic applications.

## Discussion

The use of peptides for cancer targeting is limited by their rapid degradation from proteases *in vivo*. High and repeated doses of peptides or PEGylation are thus often required to achieve sufficient delivery to the tumor site, which can negatively impact the sensitivity and safety of medical applications that use these ligands. The virus-derived A20FMDV2 peptide, despite its high affinity and specificity for the tumor-associated integrin αvβ6, is one such peptide that suffers from poor proteolytic stability, hindering its translation into the clinic for cancer imaging and therapy. Modification of A20FMDV2 to bolster its resistance against proteases is difficult because of the highly adapted and conserved structure of the peptide; its 20-amino acid sequence contains a short β sheet, followed by the tripeptide RGD motif, and then lastly a longer 3_10_ helix that together form a loop structure (38, 39). The amino acids in the β sheet and 3_10_ helix that flank the RGD motif strongly influence the affinity and specificity of A20FMDV2 for αvβ6 (57), and thus modifying these amino acids runs the risk of compromising either or both these attributes that are equally critical for the peptide's successful translation.

In this study, we synthesized a panel of more than 10 re-engineered A20FMDV2 variants that were cyclized by cysteine perfluoroarylation with DFBP and further modified with d-amino acids and nonproteinogenic analogs to improve the A20FMDV2 peptide's serum stability without sacrificing its affinity and specificity for αvβ6. From this panel, we identified three variants (DFBP-cyclized C2C18 CitTKA_D_, C2C18 P_h_ R_D_TKA_D_, and C1C18 A_D_ R_D_TK_D_A_D_) that persist in serum for ≥24 h and retain binding affinity and specificity to αvβ6^+^ cells, with the first variant demonstrating even better αvβ6 specificity than the original A20FMDV2 peptide. Given these enhanced properties, future *in vivo* usage of the cyclization and modifications reported herein could greatly improve the bioavailability of the A20FMDV2 peptide for applications concerning cancer diagnosis and treatment.

Despite our improvements to the A20FMDV2 peptide, there are opportunities for further peptide optimization and design. In our MALDI-TOF MS serum stability studies, we observed N-terminal cleavage of biotinylated Asn1 in modified C2C18 DFBP formulations as well as C-terminal cleavage at or adjacent to Thr20 for all the cyclized formulations. Hung *et al*. (58) reported that this N-terminal Asn1 and C-terminal Thr20 can be substituted with d-amino acid analogs to improve A20FMDV2 serum stability without diminishing binding affinity. They further showed that Lys16 following the RGDLXXL motif can be replaced with l-ornithine, l-2,4-diaminobutryic acid, or l-2,3-diaminopropionic acid and that Leu13 within the RGDLXXL motif can be replaced with l-2-aminoisobutryic acid or l-norvaline without reducing αvβ6 binding. Accordingly, future work combining these modifications with the cyclization and modifications described here could lead to the generation of “super-stable” A20FMDV2 peptide variants. We also speculate that finding suitable mimetics for arginine substitution in the RGD motif, while difficult, will be equally important for this goal, as cleavage of this amino acid potentially accounted for a large portion of observed degradation products in our experiments.

Another facet of our re-engineered A20FMDV2 variants that we do not explore in this study is their potential for decreased immunogenicity. Our laboratory has previously shown that repeated doses of PEG-containing micelles loaded with melittin peptides derived from bee venom can cause fatal anaphylactic reactions in immunocompetent mice that are characterized by high titers of serum IgM antibodies that bind PEG (67). Switching to micelles loaded with melittin peptides modified with d-amino acids was found to eliminate this immune hypersensitivity, suggesting that the unmodified melittin peptides served as adjuvants to induce anti-PEG antibody responses. While the A20FMDV2 peptide does not appear to be immunogenic in humans after a single microdose (42), its derivation from FMDV should raise concerns for human application, especially with PEGylation and after repeated doses. Given all this, the incorporation of d-amino acids and nonproteinogenic analogs into the A20FMDV2 sequence described in this work may also lower the potential immunogenicity of this virus-derived peptide, further increasing its safety profile for clinical applications.

## Experimental procedures

### Materials

Solvents, including *N*,*N*-dimethylformamide (DMF), dichloromethane, TFA, ethyl ether anhydrous, and ACN, were purchased from Fisher Scientific. Standard protected l-amino acids, d(+)-biotin, Fmoc-d-Ala-OH, Fmoc-d-Lys(Boc)-OH, Fmoc-d-Arg(Pbf)-OH, Fmoc-Cit-OH, Fmoc-l-Hyp(tBu)-OH, Fmoc-l-Phe(4-Boc-amino)-OH, Fmoc-l-Phe(4-Boc2-guanidino)-OH, Rink Amide resin, ethyl (hydroxyamino)cyanoacetate (Oxyma), *N*,*N*′-diisopropylcarbodiimide, piperidine, and tris-(carboxyethyl)phosphine hydrochloride were purchased from Novabiochem, AnaSpec, Chem-Impex International, and Sigma–Aldrich. 1,3-Dimethoxybenzene, triisopropylsilane, 1,2-ethanedithiol (EDT), and DFBP were purchased from Acros Organics. The QuantTag Biotin Quantification Kit was purchased from Vector Laboratories. RPMI and Dulbecco's PBS (DPBS) with magnesium and calcium were purchased from Corning. Dulbecco's modified Eagle's medium, fetal bovine serum (FBS), and StemPro Accutase were purchased from Life Technologies. Zombie Violet and Streptavidin Alexa Fluor 647 were purchased from BioLegend. Bovine serum albumin was purchased from Miltenyi Biotec. About 4% paraformaldehyde was purchased from Alfa Aesar. Normal mouse serum for stability studies was prepared in-house. Briefly, mouse whole blood was collected by cardiac puncture into BD Microtainer serum-separating tubes (Becton Dickinson) and allowed to clot for 30 min. Tubes were then centrifuged according to the manufacturer's instructions, and serum was collected and stored at −20 °C until needed. All animal handling protocols were approved by the University of Washington Institutional Animal Care and Use Committee.

### Peptide synthesis

Sequences of synthesized peptides are listed in Table S1. Peptide synthesis was performed with a Liberty Blue HT12 automated microwave peptide synthesizer (CEM) by Fmoc solid-phase peptide synthesis on a Rink Amide resin support at 0.1 mmol scale. The resin was swelled in 50:50 (v/v) DMF:dichloromethane for 20 min prior to synthesis. Deprotection of Fmoc groups occurred in 20% piperidine in DMF at 90 °C for 65 s followed by three washes with DMF. Carbodiimide chemistry was used to activate amino acids 1:2:1 molar ratio aa:*N*,*N*′-diisopropylcarbodiimide:Oxyma in DMF and couplings occurred at 90 °C for 4 min, with the exception for the first amino acid in the synthesis process, which was allowed to couple to the resin for 10 min. Double couplings were used for Fmoc-Arg(Pbf)-OH and Fmoc-d-Arg(Pbf)-OH because of the large Pbf group that sterically hinders coupling of these amino acids. After synthesis, side-chain deprotection and cleavage of the peptides from the resin was carried out in 89:5:2.5:2.5:1 (v/v) TFA:1,3-dimethoxybenzene:triisopropylsilane:EDT:H_2_O for 4 h at room temperature with end-over-end mixing. EDT was included in the cleavage cocktail only for cysteine-containing peptides. Cleaved peptides were then double precipitated in cold ethyl ether anhydrous and pelleted at 4500*g* for 5 min at 4 °C after each precipitation before drying overnight under vacuum. A small amount of cleaved product was retained and analyzed by MALDI-TOF MS (Bruker AutoFlexII) to confirm proper cleavage and deprotection of each peptide. For some of the d-arginine containing peptides, a second 1 h cleavage of the precipitated crude peptide was required because of challenges with removing the Pbf protecting group on d-arginine. Those cleavage reactions were similarly precipitated in ether before being dried overnight. The next day, crude peptides were dissolved in methanol and reprecipitated in ether to wash away remaining protecting groups. Finally, the crude peptide was dried again overnight under vacuum before HPLC purification.

### Reverse-phase HPLC purification

Crude peptides were resuspended at 80 mg/ml in 20% ACN in H_2_O containing 0.1% TFA (v/v), syringe filtered, and purified to >95% purity by reverse-phase HPLC on an Agilent 1260 Infinity equipped with a Synergi 4u Fusion-RP C18 semipreparative column (Phenomenex). Monitoring 220 nm absorbance, a flow rate of 5 ml/min and a 20 to 65% 8-min linear solvent gradient of ACN in H_2_O with 0.1% TFA were used for purification of these peptides. The molecular weights of purified peptides were confirmed by MALDI-ToF MS and were consistently within 1-2 g/mol of the expected values. After purification, organic solvent was removed by rotary evaporation and peptides were lyophilized and stored at −20 °C until further usage.

### Peptide Cyclization by Perfluoroarylation

In-solution cyclization of peptides with DFBP was conducted as previously described (54), but with some modifications. Briefly, 20 mg purified linear peptide was dissolved in 3 mL DMF with 2 molar equivalents of each TCEP and DFBP. Then, 1.5 mL 50 mM Tris base in DMF was freshly prepared and added to the reaction before vortexing (4.5 ml total). After an overnight incubation at room temperature with end-over-end mixing, the cyclization reaction was diluted in 10 ml H_2_O containing 0.1% TFA, and peptide was desalted with a Sep-Pak C18 cartridge (Waters). Peptide was eluted from the cartridge in 50:50 (v/v) ACN:H_2_O containing 0.1% TFA, and proper cyclization was confirmed by MALDI-TOF MS. Organic solvent was subsequently removed by rotary evaporation, and cyclized peptides were lyophilized and stored at −20 °C until further usage.

### Cell line culture

The K562 and A375P cell lines used in binding studies were purchased from American Type Culture Collection. The K562 αvβ6:mCherry cell line was a kind gift from Audrey Olshefsky (Pun and King Labs, University of Washington) and was generated by nucleofection of K562 cells with two linear DNA fragments, one encoding the αv integrin and a puromycin resistance gene and the other encoding the β6 integrin and a fluorescent mCherry reporter. After nucleofection, K562 αvβ6:mCherry cells were purified by puromycin selection and fluorescence-activated cell sorting. K562 and K562 αvβ6:mCherry suspension cells were cultured in RPMI1640 medium with l-glutamine and 10% FBS. Adherent A375P cells were cultured in high-glucose Dulbecco's modified Eagle's medium with l-glutamine and 10% FBS and were detached with StemPro Accutase prior to flow cytometry binding studies to preserve extracellular integrin expression.

### Flow cytometry binding studies

Biotinylated peptide stocks were prepared in H_2_O at approximately 5 mM, and the exact concentration was measured using a QuantTag Biotin Quantification Kit. Stocks were stored at 4 °C and used for binding studies within 2 weeks of preparation. Prior to binding, cells were prestained with Zombie Violet in DPBS (0.2 μl per 100 μl per 10^6^ cells) for 15 min at room temperature for dead cell discrimination. Meanwhile, peptide stocks were serially diluted in DPBS with calcium and magnesium over ice. Cells were then washed at 4 °C with DPBS 1% bovine serum albumin to neutralize the Zombie Violet, plated in a U-bottom black 96-well plate (2 × 10^5^ per well) over ice, and stained with 100 μl peptide solutions for 20 min at 4 °C. Cells were then washed twice with 200 μl cold DPBS and incubated with 100 μl streptavidin Alexa Fluor 647 in DPBS (1:500) for 20 min at 4 °C. Cells were subsequently washed twice as before and resuspended in 200 μl DPBS 0.1% paraformaldehyde for assaying on an Attune NxT Flow Cytometer (Life Technologies). Data were analyzed by FlowJo, version 10 software (Becton Dickinson), and median fluorescence intensity of singlet live cell events were used as measurements of binding. Data were normalized to A20FMDV2 binding prior to generating binding curves, apparent *K*_*D*_ values, bar graphs, and statistical testing in GraphPad Prism 6 software (GraphPad Software).

### MALDI-TOF MS serum stability

Peptides were incubated and extracted from serum as previously described (54, 55) but with some adjustments to limit serum protein carryover during extractions. Briefly, peptide stocks were diluted to 10 mg/ml in H_2_O and subsequently diluted 1:10 (v/v) in normal mouse serum for incubation at 37 °C in an incubator. At specified time points, 40 μl of the peptide/serum mixture was removed and precipitated in an equal volume of cold ACN. Precipitated serum proteins were pelleted by centrifugation at 15,000*g* for 5 min, and the supernatant with peptides (80 μl) was collected. To extract any remaining peptides, 80 μl cold 1:3 (v/v) H_2_O:ACN was added to the pellet, sonicated for 10 min, and centrifuged as before. The resulting supernatant was combined with the old one and dried under vacuum on a Savant ISS110 SpeedVac Concentrator (Thermo Fisher). The peptide pellet was then resuspended in 50 μl H_2_O and sonicated for 10 min. A small aliquot of resuspended peptide was further diluted 1:10 (v/v) in H_2_O and analyzed by MALDI-TOF MS. Resulting mass spectrums at the different time points were aligned and plotted in FlexAnalysis software (Bruker). Predicted sequences of degradation products based on product molecular weight were determined using a custom Java program called stability.jar (https://github.com/juliomarcopineda/peptide-serum-stability/releases) (55).

### LC–MS serum stability

Remaining extracted peptides from MALDI-TOF MS serum stability studies were dried as before and stored at room temperature. Within a day of LC–MS runs, pellets were resuspended in 100 μl 95:5 (v/v) H_2_O:ACN, sonicated for 10 min, and submitted for LC–MS analysis at the University of Washington School of Pharmacy Mass Spectrometry Center. Samples were injected on a TripleTOF 5600+ instrument (AB Sciex) equipped with a 2.1 × 50 mm BEH C18 column (Waters). Peptides were separated over a 5 to 100% 4-min linear solvent gradient of ACN in H_2_O with 0.1% formic acid, and peak area corresponding to the peptide molecular weight was used for quantification of remaining intact peptide. Peak areas across different time points were normalized to that of a 0 h control sample that was extracted immediately after mixing the peptide with the serum. Exponential decay curves were generated in GraphPad Prism 6 software.

## Data availability

The data that support the main findings of this study are available in this article and the supporting information. All source data generated for this study and relevant information are available from the corresponding author on reasonable request.

## Supporting information

This article contains supporting information.

## Conflict of interest

M. C. J. has interests in Umoja Biopharma and Juno Therapeutics, a Bristol-Myers Squibb company. M. C. J. is a seed investor and holds ownership equity in Umoja, serves as a member of the Umoja Joint Steering Committee, and is a Board Observer of the Umoja Board of Directors. M. C. J. holds patents, some of which are licensed to Umoja Biopharma and Juno Therapeutics. The other authors declare that they have no conflicts of interest with the contents of this article.
