# Supplementary material for: Optimized serum stability and specificity of an αvβ6 integrin-binding peptide for tumor targeting
Source: J Biol Chem. 2021 Apr 16;296:100657. doi: 10.1016/j.jbc.2021.100657 (PMC8138772; doi:10.1016/j.jbc.2021.100657)
Supplement: Table S1 and Figures S1 to S3 [file mmc1.pdf]

JBC Supporting Information

Optimized serum stability and specificity of an αvβ6 integrin-binding peptide for tumor targeting

Ian I. Cardle[1,2], Michael C. Jensen[1,2,3,4], Suzie H. Pun[1], Drew L. Sellers[1,]*

[1]*Department of Bioengineering, University of Washington, Seattle, Washington, USA*

[2]*Seattle Children's Therapeutics, Seattle, Washington, USA*

[3]*Department of Pediatrics, University of Washington, Seattle, Washington, USA*

[4]*Program in Immunology, Fred Hutchinson Cancer Research Center, Seattle, Washington, USA*

*For correspondence: Drew L. Sellers, drewfus@uw.edu

Table of Contents

Table S1. Original, cyclized, and modified A20FMDV2 peptide sequences used for binding and serum stability studies.

Figure S1. Disulfide cyclization provides less enzymatic stability than DFBP cyclization for the C1C18 peptide.

Figure S2. DFBP-cyclized C1C18 A_D R_DT$K_D$$A_D$ is degraded into a stable 138 Da smaller product over serum incubation.

Figure S3. DFBP-cyclized C2C18 peptides with arginine mimetic-modified RGD motifs fail to bind αvβ6⁺ cancer cells.

Table S1. Original, cyclized, and modified A20FMDV2 peptide sequences used for binding and serum stability studies.

Peptide	Sequence	Weight (g/mol)
A20FMDV2	biotin-NAVPNLRGDLQVLAQKVARTK-amide	2517.0
C1C18 DFBP	biotin-CAVPNLRGDLQVLAQKVCRTK-amide	2832.2
C1C19 DFBP	biotin-CAVPNLRGDLQVLAQKVACTK-amide	2747.1
C1C20 DFBP	biotin-CAVPNLRGDLQVLAQKVARCK-amide	2802.1
C2C18 DFBP	biotin-NCVPNLRGDLQVLAQKVCRTK-amide	2875.2
C2C19 DFBP	biotin-NCVPNLRGDLQVLAQKVACTK-amide	2790.1
C6C17 DFBP	biotin-NAVPNCRGDLQVLAQKCARTK-amide	2805.1
C1C18 S-S	biotin-**C**AVPNLRGDLQVLAQKV**C**RTK-amide	2536.1
C2C18 R_DTKA_D DFBP	biotin-NCVPNLRGDLQVLAQKVCR_DTKA_D-amide	2946.3
C2C18 CitTKA_D DFBP	biotin-NCVPNLRGDLQVLAQKVCCitTKA_D-amide	2947.3
C2C18 P_h R_DTKA_D DFBP	biotin-NCVP_hNLRGDLQVLAQKVCR_DTKA_D-amide	2962.3
C1C18 A_D R_DT$K_D$$A_D$ DFBP	biotin-CA_DVPNLRGDLQVLA_DQKVCR_DT$K_D$$A_D$-amide	2903.2
C2C18 $_{A}$FGD RTKA_D DFBP	biotin-NCVPNL$_{A}$FGDLQVLAQKVCRTKA_D-amide	2952.3
C2C18 CitGD DFBP	biotin-NCVPNLCitGDLQVLAQKVCRTK-amide	2876.2
C2C18 $_{G}$FGD R_DTKA_D DFBP	biotin-NCVPNL$_{G}$FGDLQVLAQKVCR_DTKA_D-amide	2994.3

C, DFBP-cyclized; **C**, disulfide-cyclized; R_D, D-arginine; A_D, D-alanine; Cit, Citrulline; P_h, hydroxyproline; K_D, D-lysine; $_{A}$F, 4-aminophenylalanine; $_{G}$F, 4-guanidinophenylalanine.

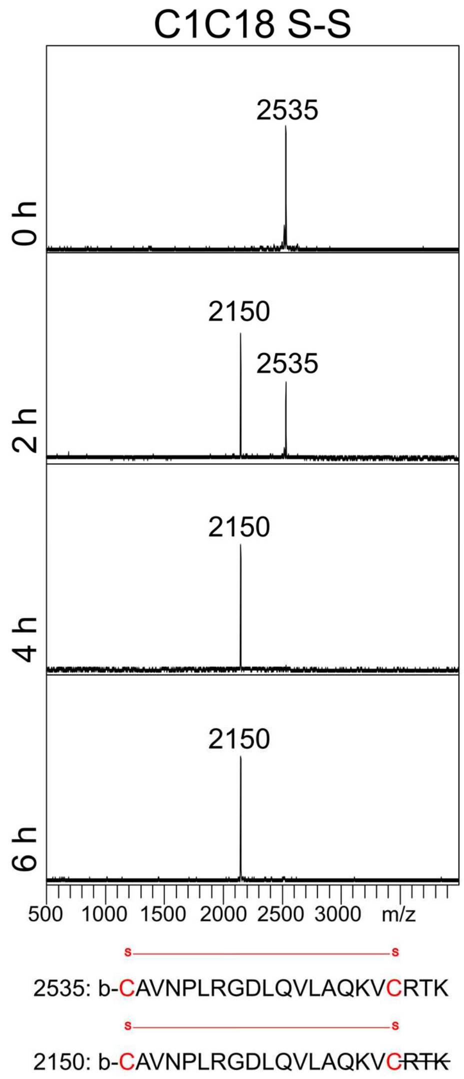

Figure S1. Disulfide cyclization provides less enzymatic stability than DFBP cyclization for the C1C18 peptide. MALDI-ToF spectra of disulfide-cyclized C1C18 S-S incubated in normal mouse serum for 0, 2, 4, and 6 h at 37 °C. Molecular weights of prominent peaks are shown. *Bottom*: predicted amino acid sequences of degradation products based on measured molecular weights.

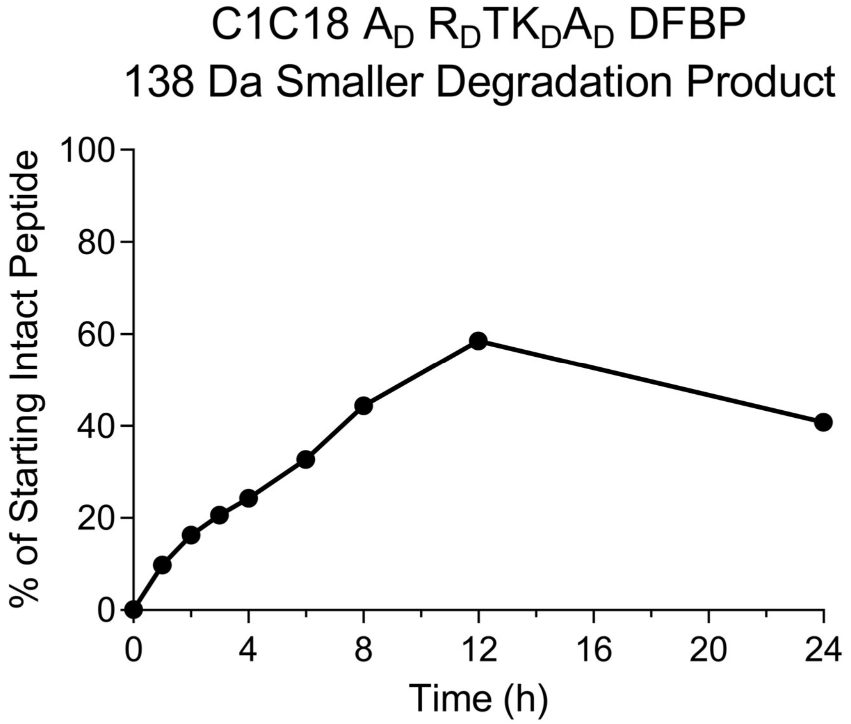

Figure S2. DFBP-cyclized C1C18 A_D $R_DTK_DA_D$ is degraded into a stable 138 Da smaller product over serum incubation. Accumulation of a 138 Da smaller degradation product from the DFBP-cyclized C1C18 A_D $R_DTK_DA_D$ peptide over a 24-h incubation in normal mouse serum, as measured by LC-MS. Values are normalized to the 0 h timepoint for the intact peptide.

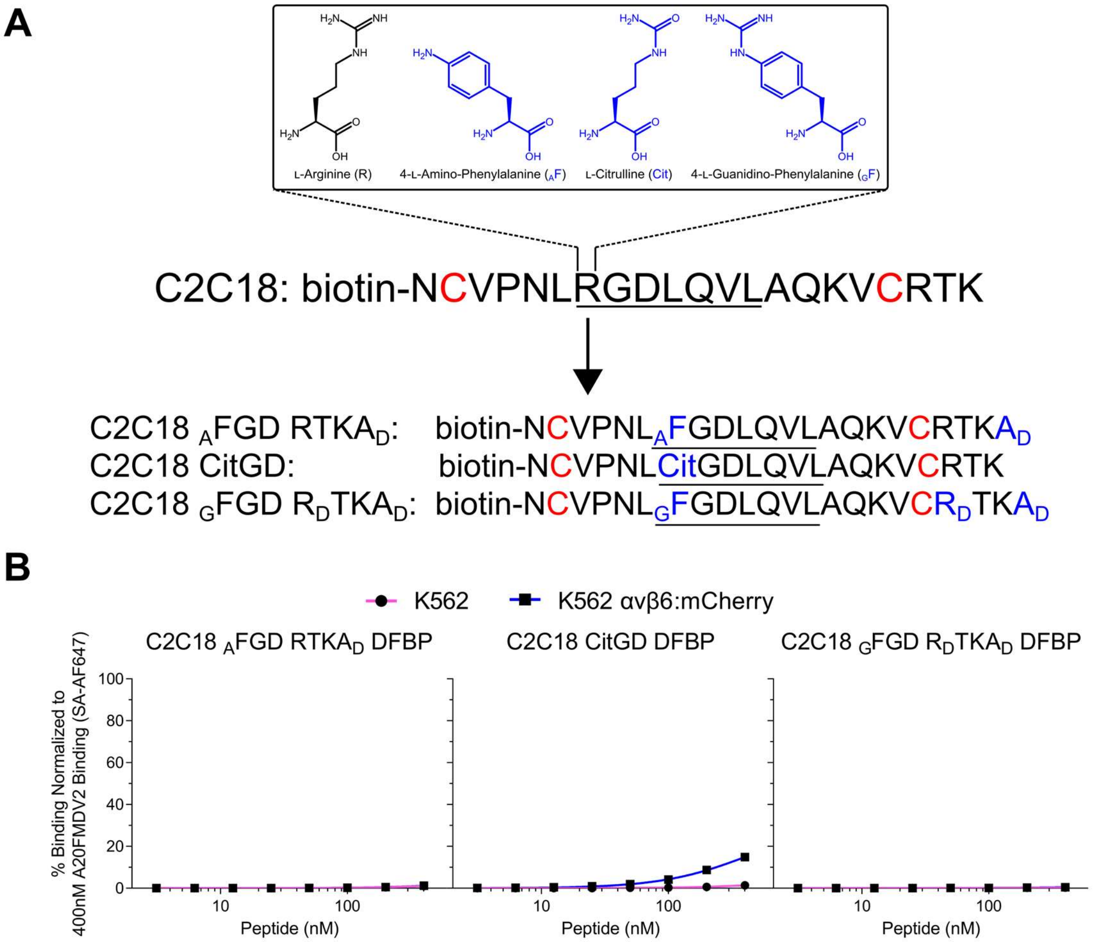

Figure S3. DFBP-cyclized C2C18 peptides with arginine mimetic-modified RGD motifs fail to bind αvβ6^{+} cancer cells. *A*, schematic of mimetic substitutions made to the sequence of C2C18 DFBP to replace arginine in the RGD motif. Chemical structures of arginine (*black*) and mimetics (*blue*) are shown for comparison. The resulting mimetic-substituted peptide sequences are also listed, with cysteine substitutions for DFBP cyclization shown in *red* and substitutions and C-terminal modifications shown in *blue*. The RGDLXXL motif that is important for αvβ6 recognition is *underlined* in all sequences. *B*, flow cytometry binding curves of mimetic-substituted peptides to K562 and K562 αvβ6:mCherry cells, normalized to 400 nM A20FMDV2 binding to K562 αvβ6:mCherry cells. The curves represent a nonlinear regression of one independent experiment in which binding data are fitted to a Hill equation. SA-AF647, streptavidin Alexa Fluor 647.
